# A manifesto for theoretical innovation

**DOI:** 10.1111/add.70440

**Published:** 2026-04-16

**Authors:** Matt Field

**Affiliations:** ^1^ School of Psychology University of Sheffield Sheffield UK

**Keywords:** decision‐making, gambling, neuroscience, recovery, relapse, treatment

*White and Kelly's article should inform the (re)imagining of a recovery‐orientated system of care for those affected by gambling‐related harm. More broadly, the article highlights opportunities to refine theories of addiction by shifting the emphasis from ‘relapse prevention’ to recovery. I argue that the psychology of decision‐making provides a useful platform for this theoretical innovation to occur*.

Although primarily focused on alcohol and other substance use disorders, White and Kelly's article [1] is particularly relevant and timely when considering support for those affected by gambling‐related harm, particularly in the UK. The first treatment guidelines were recently published [2], and the treatment and support landscape can be characterised as a mix of National Health Service (NHS), third sector and peer support organisations, with little integration [3]. The commissioning and delivery of these services is undergoing a major shake‐up, which is likely to change how services are configured and who provides them. White and Kelly's article provides an inspirational road map for what a recovery‐orientated system of support for people affected by gambling‐related harm could look like.

White and Kelly's article also challenges contemporary models of addiction to better account for addiction across its entire trajectory, from development, maintenance and persistence through to the resolution of problems and recovery. They note the ‘need to better align pre‐clinical animal research with emerging neuroscience on resilience so that such models can inform and confer greater benefit to understanding recovery’. Many models that originate from addiction neuroscience frame addiction as a chronic relapsing brain disease [4, 5, 6]. According to these models, the neuroadaptations that underlie the shift to compulsive behaviour explain why addiction persists and why relapse to substance use after a period of abstinence is the most common, if not the inevitable, outcome. These models make no serious attempt to explain how many people with addiction eventually regain control over their behaviour. As White and Kelly note, these pessimistic, deficit‐focused framings elicit counterproductive despair among people with addiction [7], and passivity among the public and professionals [8], rather than the hope that we should aim to inspire.

Given the framing of addiction as a chronic relapsing disease, treatment implications of contemporary models are typically geared towards the prevention of relapse. This often boils down to a focus on equipping people with the skills and resources that they need to navigate high‐risk situations, typically negative affect, stress and/or exposure to drug‐related cues, and the strong cravings that are triggered in those situations. Is this the best we can do? I concur with White and Kelly that we need to move beyond framing addiction as a brain disease‐related deficit that must be overcome and mitigated, towards a more positive framing of recovery capital as something to be worked towards, and that must be scaffolded by changes to contextual factors [9, 10].

This change will not happen overnight; deficit‐focused brain disease models of addiction will continue to shape research priorities and the lay understanding of addiction. However, one way to reconcile White and Kelly's approach with brain disease models is to consider how the most salient clinical implication of brain disease models (relapse prevention) could be mapped to the alternative recovery‐orientated framing. Recovery can be understood as a long‐term change to every aspect of a person's life, the journey towards which is interrupted by regularly navigating relapse‐provoking situations—sometimes successfully, sometimes not. Viewed from this perspective, the prevention of relapse remains an important treatment goal, but as brain disease models evolve, they must be able to explain how recovery occurs to the extent that the risk of relapse recedes.

One body of research that has the potential to drive innovation in this area concerns the psychology of decision‐making among people with addiction, and how decisions to use a drug (or not) are heavily constrained by context [9]. The recovery journey might be understood as a sequence of decision points characterised by exposure to relapse‐threatening situations. The outcome of each decision determines whether the person will continue on their path to recovery or experience a relapse. One theoretical model [11] expands on the crucial role of decision‐making as a determinant of why people do (or do not) relapse to addictive behaviour in high‐risk situations (see also [4]), and it also sets out what happens to the psychological machinery of decision‐making over the longer term to scaffold sustained recovery. Although in its infancy, this type of approach might bridge the gap between recovery‐orientated frameworks such as the one advanced by White and Kelly, and the enduringly influential brain disease models with their emphases on biological deficit and compulsive behaviour.

## AUTHOR CONTRIBUTIONS

**Matt Field:** Conceptualization (equal).

## DECLARATION OF INTERESTS

None.

## Data Availability

Data sharing not applicable to this article as no datasets were generated or analysed during the current study.

## References

[1] White WL , Kelly JF . On the need and contents of a specific addiction recovery research agenda. Addiction. 2026;121(7):1633–1642. 10.1111/add.70347 PMC13291095 41714158

[2] National Institute for Health and Care Excellence (NICE) . Gambling‐related harms: identification, assessment and management London: NICE; 2025 (NICE Guideline, No. 248). Available from: https://www.nice.org.uk/guidance/ng248 40106604

[3] Office for Health Improvement and Disparities . Gambling treatment: assessing the current system in England London: Department of Health and Social Care; 2024 2024. Available from: https://www.gov.uk/government/publications/gambling-treatment-assessing-the-current-system-in-england/gambling-treatment-assessing-the-current-system-in-england

[4] Heilig M , MacKillop J , Martinez D , Rehm J , Leggio L , Vanderschuren L . Addiction as a brain disease revised: Why it still matters, and the need for consilience. Neuropsychopharmacology. 2021;46(10):1715–1723. 10.1038/s41386-020-00950-y 33619327 PMC8357831

[5] Volkow ND , Koob GF , McLellan AT . Neurobiologic advances from the brain disease model of addiction. N Engl J Med. 2016;374(4):363–371. 10.1056/NEJMra1511480 26816013 PMC6135257

[6] Lüscher C , Robbins TW , Everitt BJ . The transition to compulsion in addiction. Nat Rev Neurosci. 2020;21(5):247–263. 10.1038/s41583-020-0289-z 32231315 PMC7610550

[7] Wiens TK , Walker LJ . The chronic disease concept of addiction: Helpful or harmful? Addict Res Theory. 2015;23(4):309–321. 10.3109/16066359.2014.987760

[8] Kelly JF , Greene MC , Abry A . A US national randomized study to guide how best to reduce stigma when describing drug‐related impairment in practice and policy. Addiction. 2021;116(7):1757–1767. 10.1111/add.15333 33197084 PMC8124079

[9] Acuff SF , Strickland JC , Smith K , Field M . Heterogeneity in choice models of addiction: the role of context. Psychopharmacology (Berl). 2024;241(9):1757–1769. 10.1007/s00213-024-06646-1 38990313

[10] Witkiewitz K , Tucker JA . Whole Person Recovery from Substance Use Disorder: A Call for Research Examining a Dynamic Behavioral Ecological Model of Contexts Supportive of Recovery. Addict Res Theory. 2025;33(1):1–12. 10.1080/16066359.2024.2329580 40059906 PMC11883499

[11] Field M , Murphy JG , Tucker JA , Heather N , Stafford T , Witkiewitz K . Recovery from addiction: Behavioral economics and value‐based decision making. Psychol Addict Behav. 2020;34(1):182–193. 10.1037/adb0000518 31599604

